# Case report: Surgical treatment and long-term successful outcome of a spinal intramedullary vascular malformation in a dog

**DOI:** 10.3389/fvets.2023.1243882

**Published:** 2023-08-14

**Authors:** Koen M. Santifort, Marta Plonek, Guy C. M. Grinwis, Ines Carrera, Simon Platt

**Affiliations:** ^1^Neurology, IVC Evidensia Small Animal Referral Hospital Arnhem, Arnhem, Netherlands; ^2^Neurology, IVC Evidensia Small Animal Referral Hospital Hart van Brabant, Waalwijk, Netherlands; ^3^Department of Biomedical Health Sciences, Faculty of Veterinary Medicine, Veterinary Pathology Diagnostic Centre, Utrecht University, Utrecht, Netherlands; ^4^Vet Oracle Teleradiology, Norfolk, United Kingdom

**Keywords:** hemorrhage, vascular malformation, intramedullary, hamartoma, spinal cord, outcome, prognosis

## Abstract

A 3.5-year-old male intact Staffordshire terrier crossbreed dog was presented with a one-week history of progressive paraparesis with fecal and urinary incontinence. Neurological examination was consistent with a T3-L3 myelopathy. A magnetic resonance imaging study revealed the presence of a well-circumscribed hemorrhagic space-occupying lesion at the level of T12, suspected to be a vascular malformation, such as cavernoma or arteriovenous fistula, primary hematoma or hamartoma; less likely considerations included hemorrhagic inflammation or hemorrhagic primary or secondary neoplasia. A dorsal laminectomy, durotomy, and midline dorsal myelotomy were performed with a surgical microscope, and the vascular lesion was identified and removed. Histological examination of surgical samples yielded fibrin, hemorrhage, hematoidin pigment, and some neural tissue. Although a lining wall was visualized during surgery consistent with a vascular malformation, there was no histological confirmation of such a structure, hampering definitive classification of the lesion. There was no gross or histopathological evidence that would support a diagnosis of a hamartoma or benign neoplasia. The dog was paraplegic with intact nociception the day following surgery. Ambulation was recovered within 2 weeks. Progressive and complete recovery of neurological function was seen over the next 12 weeks. No recurrence of neurological dysfunction was seen over a 12-month follow-up period. Surgical treatment should be considered in dogs with spinal intramedullary vascular lesions which can have a successful long-term outcome.

## Introduction

Central nervous system (CNS) proliferative vascular disorders include malformations (exuberant disturbances of angioarchitecture), reactive proliferations, and borderline neoplastic and neoplastic lesions (1). These can occur in the brain or spinal cord and are reported, albeit infrequently, in several animal species, including the dog, cat, cow, pig, goat and horse (1–8). Their classification has varied in the veterinary literature, but recently Marr et al. (1) categorized vascular lesions into three major groups: reactive vascular proliferations, vascular malformations including benign neoplasms (e.g., hemangioma) and hamartomas, and neoplastic disorders (e.g., hemangioblastoma, hemangiosarcoma).

In human medicine, the diagnosis of spinal vascular malformations involves an imaging screening study utilizing high-field MRI evaluating the cause of myelopathy, which is then followed up by a vascular imaging protocol to confirm the diagnosis, characterize the anatomical anomaly present, and guide treatment (9, 10). MR angiography has been shown to provide additional information regarding the type of malformation present and may help to accurately guide surgical intervention (11). If left untreated, spinal cord vascular malformations may lead to severe disablement in humans. The goal of treatment is to remove the vascular lesion while maintaining sufficient spinal cord blood supply. The treatment of choice in humans with spinal arteriovenous malformations is usually endovascular vessel obliteration (12). If this is not possible due to technical reasons, surgical ligation of the vessel is an alternative (12). MR images guide subsequent digital spinal arteriography, which is considered the gold standard diagnostic imaging technique for spinal vascular malformations. It assists with the identification of the feeding arteries, and understanding of the patient-specific vascular anatomy of the spinal cord compared to normal (13). Such diagnostic techniques are not often employed in clinical veterinary neurology/neurosurgery.

Surgical treatment of spinal intramedullary vascular lesions in dogs is sporadically reported but has been associated with good outcomes (3, 6). This case report describes the magnetic resonance imaging (MRI) features, surgical treatment and outcome of a dog with a thoracic spinal intramedullary vascular malformation.

## Case description

A 3.5-year-old intact male Staffordshire terrier crossbreed dog was presented with a 1 week history of progressive paraparesis with fecal and urinary incontinence (defecation and urination at inappropriate times, house soiling). There was no history of trauma and the dog was walked on the leash only prior to onset of signs. General clinical examination was unremarkable aside from worn nails on the digits of the pelvic limbs. The neurological examination revealed ambulatory paraparesis and ataxia with proprioceptive deficits, worse on the left side. Patellar hyperreflexia and a crossed extensor reflex were found in the pelvic limbs. A cutaneous trunci reflex cutoff was present at the level of L1. Some mild paraspinal muscular atrophy was seen at the thoracolumbar junction. Subjectively decreased sensation (or superficial nociception) was noticed in the tail and both pelvic limbs. The bladder was filled with urine and difficult to express manually, consistent with an “upper motor neuron bladder.” A T3-L3 myelopathy was suspected based on these findings. Hematology and biochemical blood tests were unremarkable. The dog was anesthetized and positioned in dorsal recumbency for a MRI study of the thoracolumbar spinal cord (1.5T Canon Vantage Elan). The following sequences were performed: T2W sagittal, T1W sagittal, STIR sagittal, STIR dorsal, T2W transverse, T1W transverse, T2* GRE transverse, T1W sagittal post-contrast, T1W transverse post-contrast. A post-scanning subtraction technique was also employed (T1W post-contrast minus T1W pre-contrast).

**Figure 1:**
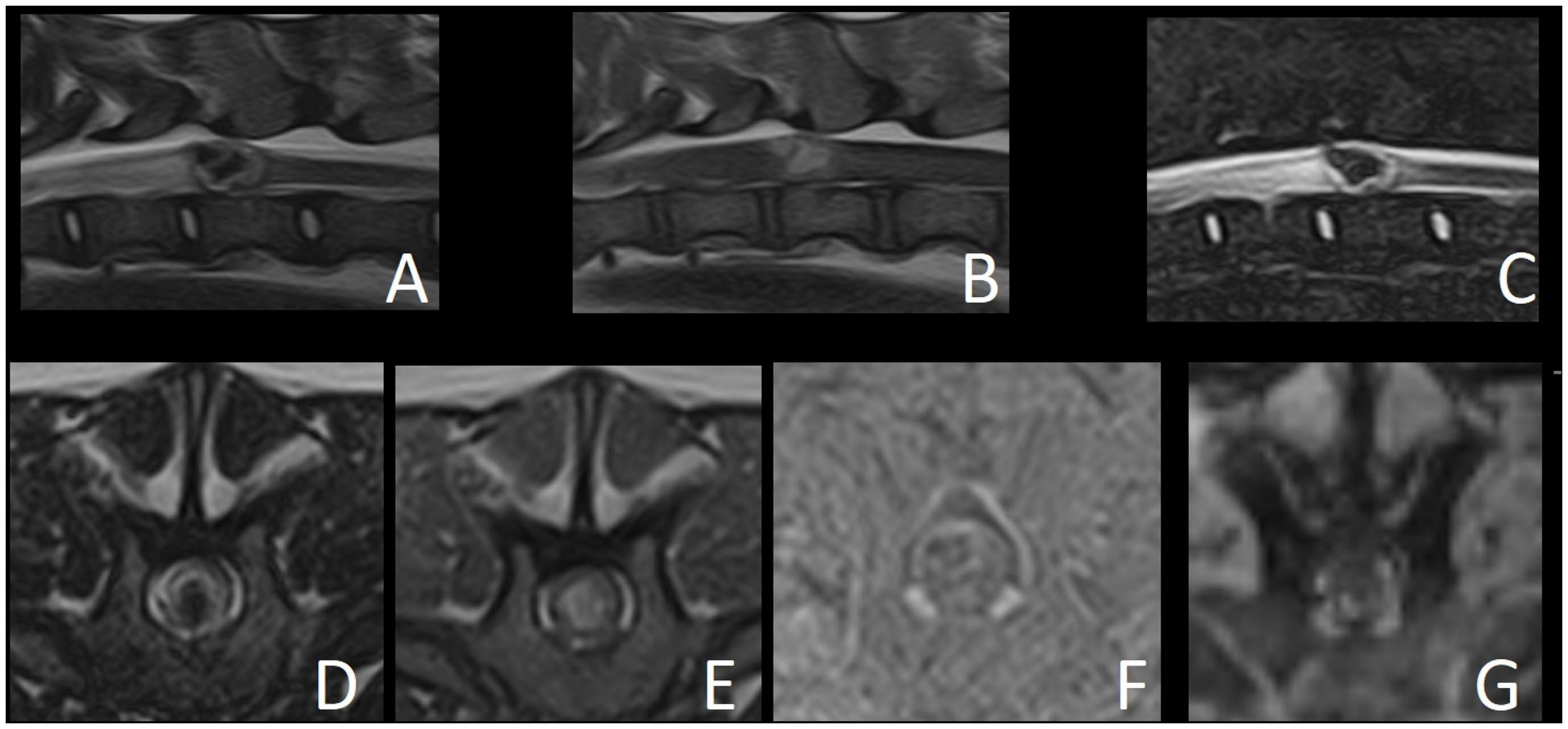
Magnetic resonance images of reported case. **(A)** T2W sagittal. **(B)** T1W sagittal. **(C)** STIR sagittal. **(D)** T2W transverse. **(E)** T1W transverse. **(F)** Subtraction transverse (T1W post-contrast minus T1W pre-contrast). **(G)** T2* GRE transverse.

The MRI study revealed a well-defined intramedullary space-occupying lesion centered and extending to the level of the vertebral body of T12 (Figure 1). The mass occupied over 75% of the cross-sectional area of the cord on its midline, with slightly more left-sided involvement. This lesion had an irregular ovoid shape, with a heterogeneous appearance in all sequences. From the T2W and STIR images, the peripheral aspect of the lesion was characterized by a very thin hypointense rim, underlined by a thicker hyperintense rim, while the center was markedly hypointense. The periphery of the lesion was slightly hypo- to isointense in T1W, whereas the center was hyperintense in T1W. This central area corresponded to a signal void in T2* sequence, suggesting the presence of hemorrhage. The lesion did not show contrast enhancement on T1W post-contrast images. The spinal cord was mildly swollen at the site of the lesion, causing obliteration of the subarachnoid space. Cranial to the intramedullary lesion, there was generalized T2W hyperintensity of the white matter, which was T1W isointense and without contrast enhancement, extending from the vertebral body of T12 to T9. Mild dilation of the central canal was also present in this segment. These findings were compatible with a pre-syrinx or spinal cord edema.

The differential diagnoses for the intramedullary space-occupying lesion at the level of T12 included a vascular malformation (such as cavernoma or arteriovenous fistula), vascular hamartoma or primary hematoma (hematomyelia). Other differentials include an inflammatory process or granuloma with hemorrhage, intramedullary neoplasia with a hemorrhagic component (such as hemangioblastoma, glioma or hemangiosarcoma) or metastatic intramedullary neoplasia. However, these seemed much less likely given the lack of contrast enhancement and the absence of a solid component. Medical treatment with prednisolone (0.3 mg/kg q12h PO – AST Beheer B.V., Oudewater, Netherlands) was initiated and further treatment options including surgical management were discussed with the owner. Neurological signs progressed over the following days to barely ambulatory paraparesis and ataxia, with increased severity of proprioceptive deficits and fecal and urinary incontinence. Surgery was performed 5 days after presentation.

**Figure 2:**
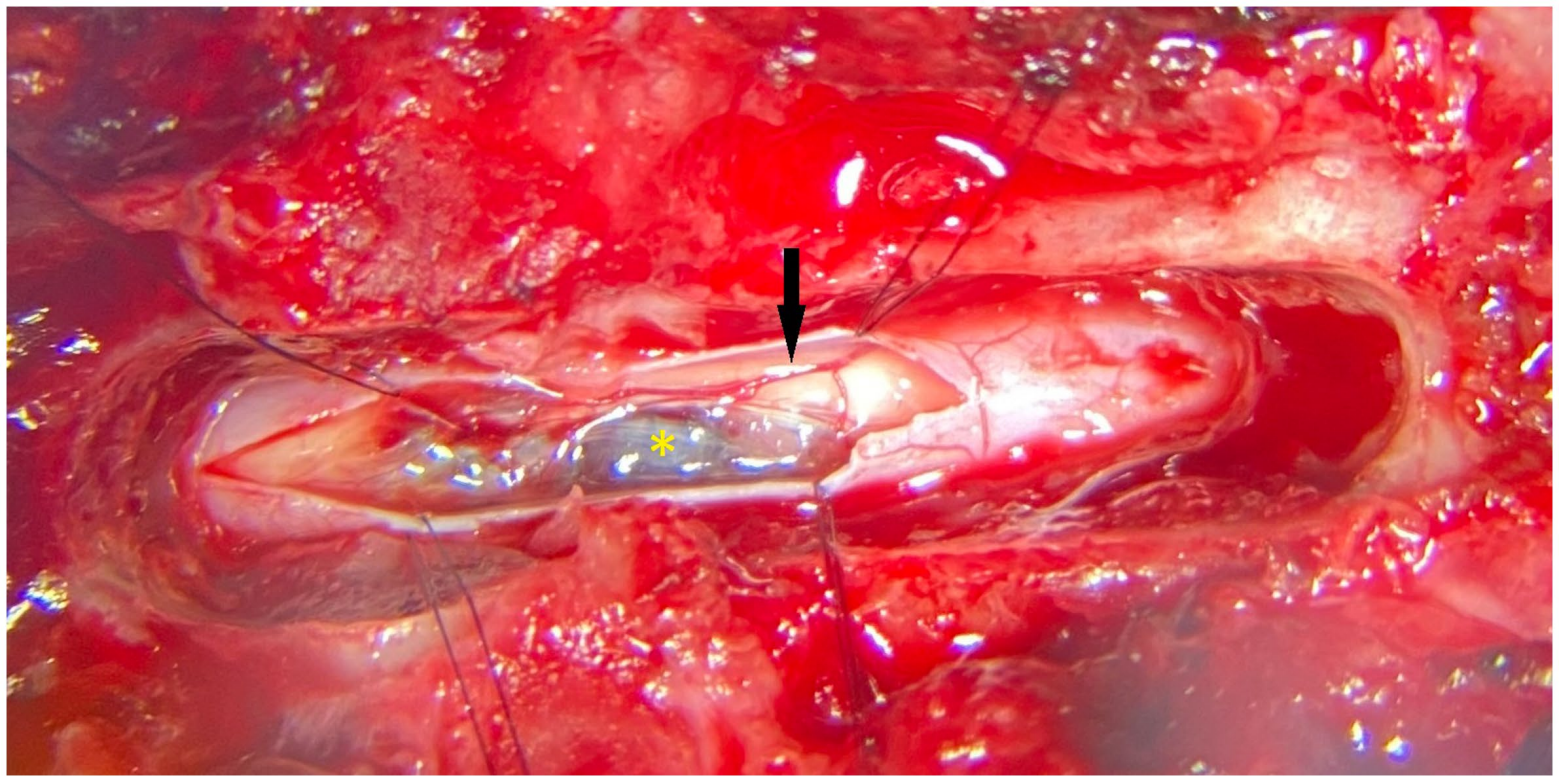
Photograph (surgical microscope) showing the intraoperative appearance (dorsal view) of the vascular malformation after dorsal laminectomy, durotomy and (partial) myelotomy. The black arrow points to the right-sided dorsal spinal artery. The yellow asterisk identifies the lesion. Cranial is to the left of the image.

The dog was anesthetized, positioned, and stabilized in sternal recumbency. Perioperative cefazoline (20 mg/kg, q6h IV – Cefazoline Mylan, Mylan B.V., Amstelveen, Netherlands) was administered. A dorsal laminectomy (T12-T13), durotomy, and midline dorsal myelotomy at the level of the dorsal median sulcus were performed with the use of a surgical microscope, and the vascular lesion in the swollen spinal cord segment was identified (Figure 2). Stay-sutures (6–0 PDS) were placed in the dura mater and arachnoid mater overlying a region of grey/blue spinal cord parenchyma and a longitudinal incision was made with an iris scalpel while retaining mild tension on the dura. At the edges of the incision, transverse cuts were performed to give an H-shaped incision and adequate exposure of the T12-T13 segments of the spinal cord after retraction and breaking of the arachnoid trabeculae. Normal vasculature of the spinal cord was visualized (e.g., dorsolaterally positioned dorsal spinal arteries and their branches). The dorsal median sulcus was evident cranially and caudally to the lesion and a midline myelotomy was made along its course. Gentle manipulation with gelatin sponges revealed the lesion. The lesion was blueish with a clear translucent wall and was somewhat adhered to grey matter on its cranial and caudal poles. The delicate wall was breached when manipulating the structure with gelatin spears and blunted nerve hooks. Next, piecemeal removal of the lesion was performed. Multiple samples were placed in buffered formalin and sent for histopathological examination. After the visually complete removal of the lesion, the spinal cord appeared reddish and devoid of some central grey matter. Hemostasis was achieved with repeated placement and removal of gelatin sponges in the cavity where the lesion was situated. Subsequently, the dura mater was closed with interrupted single sutures of absorbable monofilament 5–0. Routine closure of the epaxial musculature, fascia, subcutis and dermis was performed.

Histopathological examination of surgical samples yielded only fibrinous clots, hemorrhage, hematoidin pigment, and some (normal) neural tissue (neuronal cell bodies and glial cells). Although a vascular wall was visualized at surgery consistent with a vascular malformation, such a structure was however not found histologically, hampering the definitive classification of the lesion. Therefore, a diagnosis of a vascular malformation (not further subclassified), was concluded based on diagnostic imaging findings, surgical findings, and lack of histopathological findings supportive of reactive vascular proliferations, benign neoplasm, hamartoma or neoplasia.

Post-operative treatment consisted of urinary bladder catheterization, continued prednisolone (0.3 mg/kg q12h *per os* – AST Beheer B.V., Oudewater, Netherlands), ketamine continuous rate infusion (20–50 microgram/kg/h IV – Alfasan Nederland B.V., Woerden, Netherlands), methadone (0.3–1 mg/kg IV – Eurovet Animal Health BV, Bladel, Netherlands) based on pain score assessments, gabapentin (10 mg/kg q8h *per os* – Gabapentine Aurobindo, Aurobindo Pharma B.V., Baarn, Netherlands), omeprazole (1 mg/kg q12h *per os* – Losec MUPS, Cheplapharm Arzneimittel GmbH, Greifswald, Germany) and paracetamol (15 mg/kg q8h IV – Paracetamol Aurobindo, Aurobindo Pharma B.V., Baarn, Netherlands). The dog was paraplegic with intact nociception the day following surgery, with fecal incontinence. Over the next 2 days, the patients showed progressive return of motor function in both pelvic limbs and tail. Urinary bladder catheter was removed. Fecal and urinary continence were regained before discharge 3 days post-surgery. Medication was continued with gabapentin and prednisolone *per os*. Other analgesic medications were discontinued. Ambulation was recovered within 2 weeks. Some urination in the house was reported, but otherwise no signs of incontinence (fecal or urinary) were apparent. Gabapentin was discontinued at that point. Prednisolone was tapered off over the next 8 weeks (0.15 mg/kg q12h for 4 weeks, 0.15 mg/kg q24 h for 4 weeks). Progressive and complete recovery of neurological function was seen during the next 12 weeks. No recurrence of neurological dysfunction was seen over a 12-month follow-up.

## Discussion

This case report describes the successful outcome of a surgically treated intramedullary vascular malformation in the thoracic spinal cord of a dog. It contributes to the sporadic literature reports documenting successful outcomes of surgical treatment of intramedullary vascular malformations in dogs. Summarizing these reports, there is a chance of a good prognosis if such cases are treated surgically.

Two other case reports describe a 9-year-old Golden Retriever with a T6-7 intramedullary vascular hamartoma and a 1-year-old Labrador Retriever with a C7 intramedullary vascular malformation (3, 6). Regarding the history of these two other patients, a chronic (3 years), waxing and waning, but progressive development of clinical signs was noticed in one case, and an acute onset with progression over the next 3–6 weeks was noticed in the other (3, 6). In our case, an acute (1 week) progressive history was noted. Reasons for these differences may be manifold. Reasons for acute deterioration of neurological signs due to preexisting vascular malformation may include hemodynamic changes or hemorrhages either inside of or surrounding the vascular malformation. Surgical treatment in the previously reported cases differed from the approach in the our patient. In the first case, a lateral longitudinal myelotomy and left-sided dorsal rhizotomy of the T7 nerve rootlets were performed (3). In the second case, a sagittal durotomy and marsupialization of the dura mater flaps to the multifidus muscles were followed by a small myelotomy (6). In the former, a vascular hamartoma was diagnosed based on histopathological findings. In the latter, a vascular malformation was confirmed by the use of immunohistochemistry revealing the presence of an endothelial wall. The authors did not further subclassify the lesion but excluded some subtypes such as arteriovenous fistula (6). In another recently described case of an arteriovenous malformation in a 2-year-old crossbreed dog, surgical resection of the lesion was attempted, but discontinued due to lesion extension and the risk of intra-operative hemorrhage (8). In the case reported here, we were unable to definitively (sub)classify the vascular malformation. We were unable to confirm the presence of an endothelial wall. This may have been due to either lack of sampling of the wall during surgery (although efforts were made to include this structure) or loss of the material during processing. Still, MRI findings, intraoperative findings, long-term follow-up without recurrence of clinical signs, and the lack of histopathological findings supportive of reactive vascular proliferations, benign neoplasm, hamartoma or neoplasia, were consistent with a diagnosis of a vascular malformation.

Histopathological classification of proliferative vascular disorders in the CNS in animals has been recently reviewed (1), where vascular proliferations are classified as “reactive vascular proliferations,” “vascular malformations, benign neoplasms and hamartomas,” and “neoplastic disorders.” The authors of that review recognize the challenges veterinary pathologists are confronted with when attempting to definitively diagnose proliferative vascular disorders in the CNS. Such challenges include the overlapping histological features of the different types of lesions. Surgical incisional biopsies present a major challenge, as they rarely consist of intact lesions and are often compromised by sampling artifacts. In the case reported here, the material sent for histopathological evaluation was removed in a “piecemeal” fashion resulting in multiple tissue fragments hampering assessment and definitive classification of the lesion. However, these are “real life” issues both clinicians and pathologists have to deal with.

In the line of discussion of differential diagnoses, the authors would first like to consider human literature on the subject. In human medicine, Takai (2017) reported the most widely used classification of spinal cord vascular malformations, which divides lesions into dural arteriovenous fistulas, intramedullary glomus-type arteriovenous malformations, intramedullary juvenile-type arteriovenous malformations, perimedullary arteriovenous fistulas and extradural/paraspinal arteriovenous malformations (14). Spinal vascular malformations can also be grouped based on the differentiation between shunting versus non-shunting lesions (15). The shunting arteriovenous malformation (AVM) can be divided into type I (direct connection between an artery and one or more veins); type II (arteriovenous malformation or fistula with numerous arteries connecting to a single outflow vein); and type III (multiple arteries connecting to multiple veins) (16). Spinal cord cavernomas are intramedullary vascular malformations without an identifiable shunting vessel, that consist of enlarged thin-wall capillaries and sinusoids without spinal cord parenchyma in between the lesion (15). In human medicine, MR angiography is commonly used to differentiate the type of vascular malformation and direct the type of treatment. Of note, cavernomas are angiographically silent; however, in acute hemorrhagic cavernomas, spinal angiography is recommended to identify a small glomerular AVM that can go unnoticed on spinal MRI (15). The MRI appearance of a cavernoma is typically described as a well-defined lesion with a “popcorn ball” shape. Depending on the time of the intralesional hemorrhage, cavernomas may show different signal intensities, reflecting the degradation products of the hemoglobin (17). The MRI findings described in this case report mirror the appearance of a cavernoma, and this is why it was included as a main differential diagnosis, being a subclassification of vascular malformation.

Vascular malformations are also considered hamartomas. Hamartomas are characterized by non-neoplastic proliferation or an improper proportion of cells that are normally present in the involved tissue (e.g., blood vessels, interstitial cells, or organ-specific cell types); they are the result of aberrant development of normal tissue (18). Indeed, the term “vascular hamartoma” is often employed in veterinary literature (1, 19, 20). However, a recent veterinary review on the classification of proliferative vascular disorders of the CNS recommended abandoning the use of this term (1), because unique histopathological features are lacking. Therefore, the authors of this case report opted to use the more descriptive and general term of “vascular malformation.”

Finally, regarding differential diagnoses, tumors such as hemangioblastoma, astrocytomas or ependymomas may have intralesional hemorrhage. However, they normally show a solid portion and some degree of contrast enhancement, which both were lacking in the patient reported here. In addition to the lack of typical imaging findings, no solid mass lesion was found during surgery and no neoplastic cells were found in samples submitted for histopathology.

Surgical treatment of intramedullary mass lesions is described in veterinary literature for various pathologies, including vascular lesions, various anomalies (e.g., syringes, cysts) and neoplasia (3, 6, 21, 22). Human literature specifically covering the surgery of vascular malformations is available in plenty, while literature pertaining to clinical application in dogs (other than case reports) is lacking (23, 24). Ideally, the surgical approach to a suspected intramedullary vascular malformation requires a detailed understanding of the normal vascular anatomy of the spinal cord along with advanced utilization of MR techniques and digital angiography which will enable precise understanding of the anatomical variation present in the individual case (9–13). With the exception of case reports, there are few studies describing angiography of the spinal cord and associated structures in veterinary medicine, mostly employing computed tomographic (CT) angiography. One study described the morphometry of the lumbar internal vertebral venous plexus in dogs making use of CT angiography (25). A recent study describing the use of fast three-dimensional contrast-enhanced magnetic resonance angiography of the canine lumbar spinal cord vascular supply was unable to visualize most of the small vessels of the spinal cord, though none of the included dogs were diagnosed with vascular malformations or abnormalities (26). A study using CT angiography and 4-D magnetic resonance angiography showed that MRI allowed better identification of blood flow dynamics than CT angiography for the diagnosis of subclavian steal syndrome in a dog (27). CT was used in combination with MR in three cats with arteriovenous fistulas in the thoracic region of the spinal cord. The authors found that both imaging modalities complemented one another to reach a diagnosis (28). An angiographic study was not performed in the case reported here. Possibly, such a study could have provided more information leading up to the surgery.

In conclusion, we report the surgical treatment and associated good long-term outcome of a thoracic spinal intramedullary vascular malformation in a dog. Surgical treatment should be considered in dogs with spinal intramedullary vascular lesions and can have a good long-term prognosis.

## Data availability statement

The original contributions presented in the study are included in the article/supplementary material, further inquiries can be directed to the corresponding author.

## Ethics statement

Ethical approval was not required for the studies involving animals in accordance with the local legislation and institutional requirements because the animal was treated in accordance with the local legislation and institutional requirements. Written informed consent was obtained from the owners for the participation of their animals in this study.

## Author contributions

KS and MP performed the surgery and clinically managed the case. KS, MP, SP, and IC interpreted and reviewed MRI findings. GG performed histopathological examinations. KS wrote the first draft of the manuscript. KS, MP, GG, IC, and SP participated in the revision of the manuscript. All authors read, commented on, and approved the final manuscript.

## Funding

This publication fee was covered by IVC Evidensia’s fund for publication of peer-reviewed scientific articles.

## Conflict of interest

The authors declare that the research was conducted in the absence of any commercial or financial relationships that could be construed as a potential conflict of interest.

## Publisher’s note

All claims expressed in this article are solely those of the authors and do not necessarily represent those of their affiliated organizations, or those of the publisher, the editors and the reviewers. Any product that may be evaluated in this article, or claim that may be made by its manufacturer, is not guaranteed or endorsed by the publisher.
